# Role of Oxidative Stress in Liver Health and Disease

**DOI:** 10.1155/2016/9037051

**Published:** 2016-03-22

**Authors:** Pablo Muriel, Karina R. Gordillo

**Affiliations:** ^1^Laboratory of Experimental Hepatology, Department of Pharmacology, CINVESTAV-IPN, Avenida Instituto Politécnico Nacional 2508, Colonia San Pedro Zacatenco 07360, Apartado Postal 14-740, 07000 México, D.F., Mexico; ^2^Lipid Research Laboratory, VA Medical Center, 50 Irving Street NW, Washington, DC 20422, USA; ^3^Department of Biochemistry and Molecular Medicine, The George Washington University, 2300 I Street NW, Suite 530, Washington, DC 20037, USA

Liver diseases are a worldwide medical problem because the liver is the principal detoxifying organ and maintains metabolic homeostasis. The liver metabolizes various compounds that produce reactive oxygen radicals (ROS). Prooxidants are ROS which can cause tissue liver damage and whose levels may be increased by certain drugs, infection, external exposures, tissue injury, and so forth. Oxidative stress can result from an increase in prooxidant formation or a decrease or deficiency in antioxidants. Molecular redox switches and oxygen sensing by the thiol redox proteome and by NAD/NADP and phosphorylation/dephosphorylation systems are bias involved in signaling, control, and balance redox of a the liver system.

Because of their reactivity, ROS readily interact with all cellular macromolecules. ROS cleave the phosphodiester bonds holding bases in RNA and DNA together, breaking the chain structure of RNA and DNA. Polyunsaturated fatty acids are also a major target for oxidation by ROS, in a process called lipid peroxidation that disrupts normal membrane structure leading to necrosis. In addition, ROS, especially the hydroxyl radical, oxidize the SH group of cysteine residues of proteins to the disulfides or to the sulfoxide or the sulfonic acid; since enzymatic activity depends on cysteine, enzymes are inactivated by ROS. Also oxidative stress contributes to fibrogenesis by increasing harmful cytokines such as transforming grown factor-*β* (TGF-*β*), interleukin-6 (IL-6), and tumor necrosis factor-*α* (TNF-*α*).

However, ROS are not always the bad guy; important transcription factors such as Nrf2, NF-kB, and AP-1 are activated by H_2_O_2_-dependent oxidation of thiol residues. These transcription factors subsequently activate many genes, some of which code for cellular antioxidants. Thus, low levels of ROS can cover up for high levels of ROS. Endogenous (e.g., glutathione) or exogenous antioxidants (mainly from diet) inhibit either formation of ROS or remove/scavenge the generated radicals. Due to the central role of oxidative stress on liver disease, this special issue was devoted to its implication in hepatic health and disease.

From Mexico, Dr. J. Camacho et al. (in “Ion Channels and Oxidative Stress as a Potential Link for the Diagnosis or Treatment of Liver Diseases”) reviewed the link of ion channels and oxidative stress in hepatic injury of various etiologies; the main conclusion is that such association may be useful to develop new treatments for the principal liver diseases. The group of Dr. H.-S. Lee et al. from Taiwan (in “Sympathetic Nervous System Control of Carbon Tetrachloride-Induced Oxidative Stress in Liver through *α*-Adrenergic Signaling”) found that the sympathetic nervous system allows oxidative stress to damage the liver, thus suggesting that targeting the hepatic *α*-adrenergic signaling may provide a therapeutic approach to fight liver disease. Obesity associated with excessive alcohol consumption produces fatty liver; in this regard. Professor M.-C. Gutiérrez-Ruiz et al. from Mexico (in “Cholesterol Enhances the Toxic Effect of Ethanol and Acetaldehyde in Primary Mouse Hepatocytes”) found that the combination of ethanol and cholesterol in vitro produced a potent damage in steatotic hepatocyte. From Italy, Professor A. Galli et al. (in “Oxidative Stress in the Healthy and Wounded Hepatocyte: A cellular Organelles Perspective”) provided us with a very original review about the evolving concept of oxidative stress in the cellular hepatocyte compartments, highlighting the essential mechanisms of damage caused by free radicals.

The protective effect of dietary curcumin against alcohol induced liver disease and atherosclerosis was reported by the group of Professor M. R. Lakshman et al. from Washington (in “Protective Role of Dietary Curcumin in the Prevention of the Oxidative Stress Induced by Chronic Alcohol with respect to Hepatic Injury and Anti-atherogenic Markers”). Since ischemia/reperfusion (IR) injury is still an unsolved problem in the clinical practice, efforts are being made to prevent it; in this regard, Dr. P. C. Pérez et al. from Mexico demonstrated that spironolactone reduced liver damage produced by IR by increasing IL-6 production and catalase activity (“Spironolactone Effect in Hepatic Ischemia/Reperfusion Injury in Wistar Rats”). Oligonol, a low molecular weight polyphenol derived from lychee fruit, was reported by Dr. J.-O. Moon et al., from Korea, to ameliorate CCl_4_-induced liver injury by antioxidant effects decreasing NF-*κ*B activation via blockade of the MAPKs and Akt kinases (“Oligonol Ameliorates CCl_4_-Induced Liver Injury in Rats via the NF-Kappa B and MAPK Signaling Pathways”). In the review made by Professor R. Hernández-Muñoz et al. from Mexico (“Is Liver Enzyme Release Really Associated with Cell Necrosis Induced by Oxidant Stress?”), the utility of liver enzymes as markers of oxidative stress is challenged. Dr. Li et al., from Taiwan (“The Protective Effects of Trypsin Inhibitor on Hepatic Ischemia-Reperfusion Injury and Liver Graft Survival”), studied the effect of ulinastatin on liver IRI and graft survival in mice and found that this compound affords significant protection to donor livers from cold IRI, probably by inhibition of proinflammatory cytokine release and modulating apoptosis.

These authors highlight both the importance of free radicals in the development, establishment and perpetuation of liver disease, and the potential therapeutic effect of compounds that directly or indirectly interfere with the prooxidant process.

Hopefully, this publication will provide a benchmark for future investigations evaluating a far greater body of basic and clinical evidence regarding the role of oxidative stress in liver health and disease as well the beneficial effect of antioxidant therapy to prevent or reverse hepatic injury.

*Pablo Muriel* *Pablo Muriel* *Karina R. Gordillo* *Karina R. Gordillo*

